# Development of human mycoplasma vaccines: navigating the IL-17A paradox

**DOI:** 10.3389/fimmu.2025.1720943

**Published:** 2025-12-08

**Authors:** Wenjun Zhang, Tingting Li, Fangyi Guo

**Affiliations:** 1 The First People’s Hospital of Chenzhou, First Clinical College, Xiangnan University, Chenzhou, China; 2 Changde Hospital, Xiangya School of Medicine, Central South University (The First People’s Hospital of Changde City), Changde, China

**Keywords:** mycoplasmas, interleukin-17A (IL-17A), vaccines, vaccine-enhanced disease (VED), antibody

## Abstract

Despite decades of effort, effective vaccines against pathogenic human mycoplasmas remain elusive, largely due to vaccine-enhanced disease (VED). Recent discoveries highlight the paradoxical role of interleukin-17A (IL-17A) as both protector and instigator of pathology. Although IL-17A is indispensable for mucosal defense against many pathogens, its dysregulated expression following mycoplasma vaccination can provoke excessive neutrophilic inflammation and exacerbate pulmonary injury. This review synthesizes emerging evidence implicating bacterial lipoproteins as key triggers of maladaptive IL-17A responses. We delineate the duality of IL-17A in mycoplasma infections, mediating both protection and tissue damage, and critically examine its implications for vaccine design. Integrating insights from recent animal models, B-cell depletion studies, and proteomic analyses, we propose a framework for next-generation vaccines that elicit protective immunity while circumventing IL-17A-driven immunopathology.

## 1 Introduction

*Mycoplasma* sp*ecies* exemplify evolutionary minimalism, representing highly specialized pathogens that have undergone extensive reductive evolution, retaining only genes essential for survival (1). This extreme genomic streamlining has produced some of the smallest genomes among self-replicating organisms, imposing profound metabolic constraints. Lacking key biosynthetic pathways, mycoplasmas must scavenge vital nutrients, including amino acids, cholesterol, and folate, directly from host cells to complete their life cycle, rendering them strict obligate parasites (2, 3). To sustain this parasitic lifestyle, they have evolved sophisticated multiprotein adhesion complexes containing specialized adhesins that mediate strong attachment to host respiratory and urogenital epithelia (4). This exceptional adhesion capacity underlies their frequent association with acute respiratory diseases and urogenital tract infections in humans (5–8).

The clinical management of mycoplasma infections remains increasingly challenging. Current therapeutic options are limited, with macrolide antibiotics, such as erythromycin and azithromycin, constituting the principal line of treatment (9–11). However, the emergence and global dissemination of macrolide- and fluoroquinolone-resistant strains across multiple mycoplasma species have led to frequent treatment failures and recurrent infections, complicating patient care (10, 12, 13). The clinical management of mycoplasma infections remains increasingly challenging. Current therapeutic options are limited, with macrolide antibiotics such as erythromycin and azithromycin constituting the principal line of treatment. This growing antimicrobial resistance crisis, combined with the substantial public health burden imposed by mycoplasmas, underscores the urgent need for effective prophylactic vaccines. Mycoplasma infections contribute significantly to global morbidity and economic loss. *M. pneumoniae* is a leading cause of community-acquired pneumonia, resulting in a substantial number of infections and hospitalizations annually (14, 15). Severe infections can progress to refractory or necrotizing pneumonia, presenting with respiratory distress, pleural effusion, and potentially fatal respiratory or circulatory failure (16, 17). Extrapulmonary complications encompass a range of serious conditions, with considerable mortality rates in vulnerable populations (18, 19). *M. genitalium* is frequently detected in cases of non-gonococcal urethritis and pelvic inflammatory disease, and can cause chronic sequelae such as tubal factor infertility and ectopic pregnancy (20–23). The direct and indirect healthcare costs associated with mycoplasma infections impose a significant economic burden (24, 25). Despite decades of research, no vaccine against human-pathogenic mycoplasmas has achieved regulatory approval (26–28), largely due to the risk of vaccine-enhanced disease (VED) (28). Understanding the mechanistic basis and historical challenges underlying this phenomenon is therefore critical to advancing next-generation vaccine development.

## 2 The historical challenge of mycoplasma VED

The pursuit of mycoplasma vaccines began in the 1960s with pioneering trials using formalin-inactivated *M. pneumoniae* whole-cell preparations in human volunteers. Unexpectedly, a substantial proportion of vaccinated individuals exhibited more severe clinical manifestations upon natural infection compared with unvaccinated controls (29, 30). This paradoxical outcome, later termed VED, represented a serious and unforeseen consequence that led to the immediate termination of human trials (31).

Subsequent animal studies have consistently reproduced and expanded upon these findings. Vaccination with diverse experimental platforms, including live-attenuated strains, killed whole-cell preparations, and crude protein extracts, followed by pathogen challenge, reliably induced exacerbated pulmonary pathology characterized by dense inflammatory infiltrates and increased tissue damage (32–34). These reproducible outcomes, often accompanied by markedly elevated interleukin-17A (IL-17A) expression and massive neutrophil accumulation, firmly established VED as the central obstacle to the successful development of mycoplasma vaccines (31, 35).

Recognizing the limitations of whole-cell vaccine approaches, subsequent research shifted toward next-generation platforms. Subunit and nucleic acid-based vaccines (DNA and mRNA) offered theoretical advantages, including improved safety profiles, precise antigenic targeting, and reduced risk of non-specific immune activation (26, 27, 36, 37). However, critical barriers persist: production costs remain prohibitively high, manufacturing processes are technically demanding, and, most importantly, vaccine-enhanced disease (VED) continues to emerge across multiple platforms (25, 38). This recurring failure across distinct vaccine modalities underscores the urgent need for deeper mechanistic insight into the immunopathological processes driving VED (39, 40).

## 3 The dual nature of IL-17A in mucosal immunity

Interleukin-17A (IL-17A) is a pleiotropic, pro-inflammatory cytokine primarily secreted by T helper 17 (Th17) cells, a specialized CD4^+^ subset, alongside other immune cell types such as γδ T cells, innate lymphoid cells (ILCs), and natural killer T (NKT) cells (41). IL-17A plays a pivotal role in mucosal immunity: murine studies demonstrate its importance in clearing a wide range of pathogens, including bacteria, fungi, and parasites (42–44). Consistent with these findings, humans with genetic defects that impair Th17 differentiation exhibit marked susceptibility to mucosal infections caused by Staphylococcus aureus and Streptococcus pneumoniae (42, 45).

Harnessing IL-17A has proven beneficial in vaccine development against various pathogens. Distinct strategies can elicit IL-17-mediated protection through diverse mechanisms. For example, the CAF01 adjuvant, which strongly induces IL-17A responses, enhances immunity against *Staphylococcus aureus* (46). Similarly, glycolipid-coated liposomal vaccines confer mucosal protection against streptococcal infections in animal models through robust IL-17A induction (47). Other adjuvants share this capacity: LTA1, derived from the A1 domain of *E. coli* heat-labile enterotoxin, generates lung-resident Th17 cells following intrapulmonary administration, conferring protection against *Klebsiella pneumoniae* (48, 49). The route of vaccine administration also critically shapes IL-17-driven immunity. Mucosal delivery routes such as intranasal or intratracheal vaccination preferentially induce potent pulmonary Th17 responses that provide superior protection against respiratory pathogens like *Mycobacterium tuberculosis*. Notably, this protection is IL-17-dependent but IFNγ-independent (50). These mucosal Th17 cells strategically localize within inducible bronchus-associated lymphoid tissue via IL-17–dependent CXCL13 induction, enhancing macrophage activation and pathogen clearance (50). However, IL-17A’s role is highly context dependent; in mycoplasma infections, it exhibits paradoxical and often pathogenic effects (39, 40).

## 4 IL-17A as a mediator of mycoplasma VED

In *Mycoplasma* infections, IL-17A consistently displays pathogenic potential across multiple clinical contexts. For instance, ureaplasma-induced IL-17A production has been implicated in respiratory and neurological complications in preterm neonates (51), while *Ureaplasma urealyticum* infection elevates seminal IL-17A levels, impairing sperm quality and contributing to male infertility (52). The role of IL-17A in *Mycoplasma pneumoniae* infection, however, is paradoxical and complex. Clinical data reveal inconsistent correlations between IL-17A levels and disease severity: some studies associate elevated serum IL-17A with severe pneumonia and adverse outcomes (53), whereas Yang et al. found that reduced IL-17A levels predicted more severe, refractory disease in children (54). Complementary findings by Fan et al. identified discordant IL-17A distribution, lower serum but higher bronchoalveolar lavage fluid (BALF) levels, in severe cases, suggesting compartmentalized immune responses (55). Collectively, these conflicting observations highlight IL-17A’s dualistic role and emphasize the need for caution in vaccine design, as the threshold separating protective from pathological IL-17A responses remains undefined. Experimental models provide more definitive evidence: immunization with live-attenuated *M. pneumoniae* or lipoprotein-rich subunit vaccines (LAMPs) induces pronounced pulmonary injury upon challenge, with histopathological severity directly proportional to IL-17A levels (39, 40).

## 5 Mechanisms of VED: dissecting the roles of lipoproteins, IL-17A, neutrophils, and B cells

**Figure 1:**
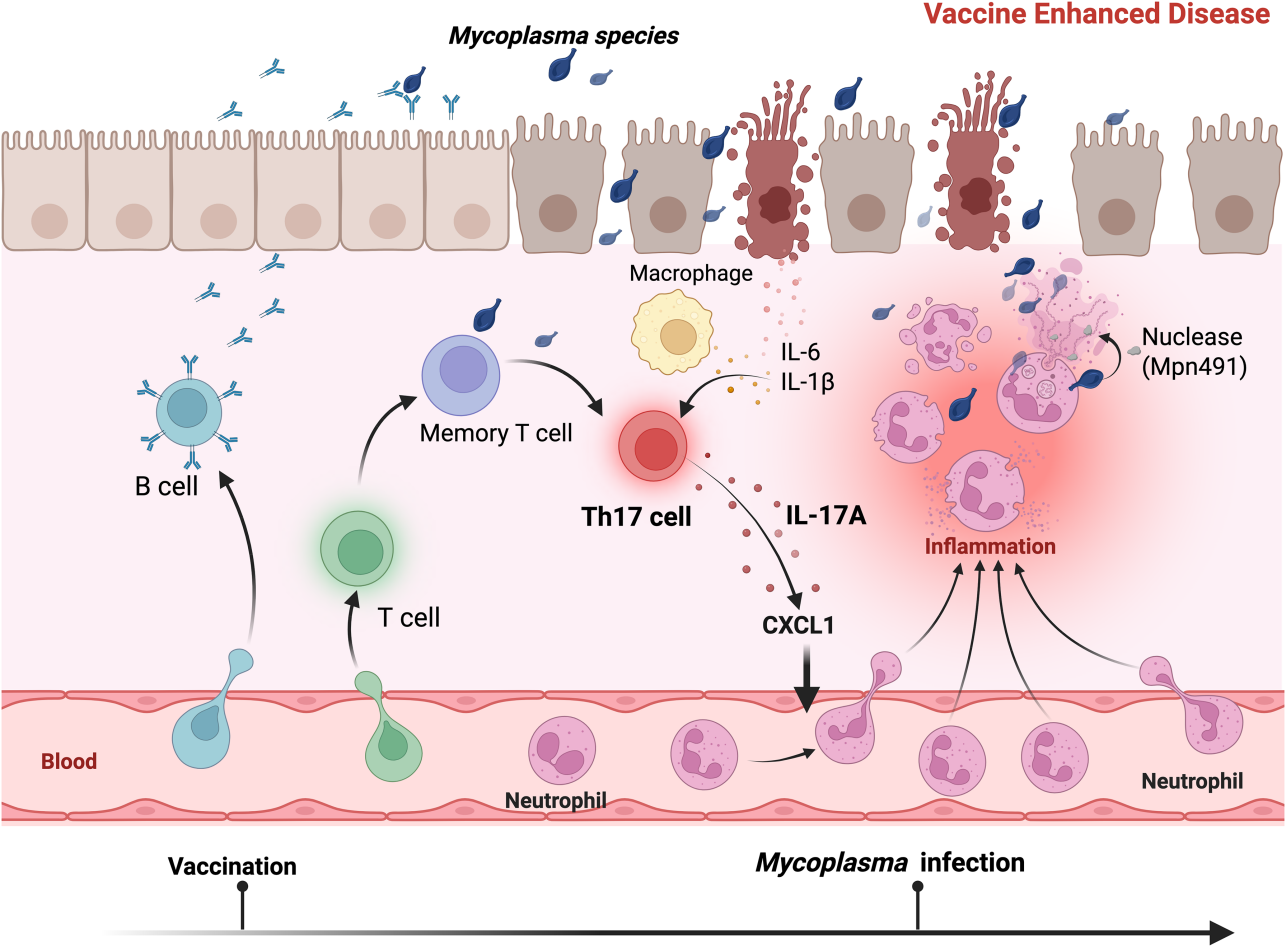
Mechanistic overview of vaccine-enhanced disease in *Mycoplasma* infection. Vaccination primes adaptive immune responses including B cells, memory T cells, and conventional T cells. Upon subsequent *Mycoplasma* challenge, memory responses are recalled with pathological Th17 cell differentiation and IL-17A overproduction. IL-17A drives excessive secretion of the neutrophil chemoattractant CXCL1, resulting in massive neutrophil recruitment to infected airways. Despite florid neutrophilia, *Mycoplasma* evades neutrophil-mediated killing through secretion of nuclease (Mpn491) that degrades neutrophil extracellular traps. The unopposed neutrophil infiltration causes severe tissue damage without effective bacterial clearance, manifesting as vaccine-enhanced immunopathology. IL-6 and IL-1β contribute to Th17 polarization. B cells play a dual role: they provide partial protection through antibody-mediated reduction of bacterial burden while serving critical immunoregulatory functions that constrain excessive inflammation. The balance between protective humoral immunity and pathological cell-mediated responses determines disease outcome following vaccination and challenge. Created in BioRender. https://BioRender.com.

Mechanistically, mycoplasma-associated VED differs fundamentally from other well-characterized forms of vaccine-enhanced pathology. In diseases such as dengue (56), respiratory syncytial virus (57), and measles (58), enhancement arises predominantly through antibody-dependent enhancement (ADE). In contrast, mycoplasma VED operates through a distinct, multifaceted mechanism involving intricate crosstalk between innate and adaptive immune pathways (**Figure 1**).

### 5.1 Disease severity in mycoplasma reinfection

Evidence from both animal and human studies indicates that repeated mycoplasma exposure can paradoxically induce immune-enhanced disease rather than protective immunity. In murine models, *M. pneumoniae* reinfection elicits accelerated inflammatory responses within three days, compared to 10–14 days for primary infection, driven by CD4^+^ T cells that produce 10- to 100-fold elevations in proinflammatory cytokines and dense perivascular infiltrates. Even heat-killed organisms provoke these responses, confirming the role of immunological memory (59–61). In humans, naturally acquired infection generates only partial and transient protection: individuals who develop pneumonia during primary infection maintain elevated antibody titers and reduced reinfection rates for 2–9 years, whereas those with mild or subclinical disease lose immunity rapidly (62). The observation that childhood infections are typically mild but later exposures more often lead to pneumonia suggests that repeated infections progressively prime hyper-inflammatory responses. This conclusion is reinforced by findings that immunosuppressed hosts exhibit reduced pathology despite equivalent bacterial replication, whereas immunocompetent individuals mount exaggerated, tissue-damaging inflammation (63).

These insights carry critical implications for vaccine design. Formulations that strongly prime cellular immunity without preventing colonization may inadvertently predispose recipients to vaccine-enhanced disease (VED) upon re-exposure. Unlike classical antibody-dependent enhancement, this mechanism reflects T cell–mediated immunopathology rather than antibody-mediated pathogen entry. A mechanistic understanding of these immune pathways is therefore essential to achieving vaccines that elicit durable protection while avoiding hyper-inflammatory outcomes.

### 5.2 Lipoproteins as the primary instigators

A pivotal breakthrough identified *Mycoplasma* lipoproteins as the primary mediators of VED. Vaccination with lipid-associated membrane proteins (LAMPs) reproducibly induces VED, whereas enzymatic delipidation (dLAMPs) abolishes pathology (39).The lipid moieties act as potent Toll-like receptor (TLR) agonists, engaging TLR2/1 and TLR2/6 heterodimers that recognize triacylated and diacylated lipoproteins, respectively (64). This TLR signaling profoundly skews T cell differentiation toward pathogenic Th17 polarization while suppressing regulatory T cell development (39, 65).

### 5.3 IL-17A–neutrophil axis of tissue destruction and interconnected cytokine networks in VED

#### 5.3.1 Paradoxical roles of IL-17A in *Mycoplasma* VED

Upon pathogen challenge, lipoprotein-primed hosts mount an exaggerated anamnestic response dominated by IL-17A overproduction and excessive secretion of the neutrophil chemoattractant KC (CXCL1), resulting in severe pulmonary neutrophilia (40). Notably, *M. pneumoniae* is intrinsically resistant to neutrophil-mediated killing, partly due to a secreted nuclease (Mpn491) that degrades neutrophil extracellular traps (66, 67). Consequently, the intense neutrophil infiltration becomes largely pathologic, causing tissue injury without reducing bacterial load. Therapeutic IL-17A neutralization or neutrophil depletion markedly alleviates lung pathology, definitively establishing their causal role in VED (40).

However, IL-17A plays a dual role in mycoplasma immunity, contributing to both protection and pathology depending on its timing, magnitude, and immunologic context. Early, moderate IL-17A responses may aid bacterial clearance by recruiting neutrophils and maintaining epithelial barrier integrity (40, 41), whereas excessive or sustained IL-17A production, such as that induced by LAMP vaccination, overwhelms these benefits and drives immunopathology (40). This dose- and context-dependent duality remains poorly defined, warranting investigation into kinetic thresholds that distinguish protective from pathological IL-17A activity.

#### 5.3.2 IL-6 and IL-1β: amplifiers of the Th17 response

The pathogenic role of IL-17A in mycoplasma VED implicates upstream cytokines that promote Th17 differentiation, particularly IL-6 and IL-1β (40, 68, 69). In addition to priming an exaggerated IL-17A response, *M. pneumoniae* LAMPs also upregulate the production of IL-6 and IL-1β upon challenge (40). These cytokines serve dual roles as both inflammatory mediators and key drivers of Th17 cell differentiation and maintenance. IL-1β, together with IL-6 and TGF-β, initiates Th17 lineage commitment (70, 71), while IL-6 simultaneously suppresses regulatory T cell (Treg) development, removing a critical constraint on inflammation (72).

The contributions of IL-6 and IL-1β to VED likely extend beyond Th17 amplification. IL-6 acts as a pleiotropic cytokine that triggers acute-phase responses, enhances neutrophil survival, and sustains inflammatory cascades (73). IL-1β further aggravates disease by directly activating endothelial cells and increasing vascular permeability, thereby promoting pulmonary edema and leukocyte extravasation (74). Collectively, these mediators may form a self-amplifying inflammatory loop: following mycoplasma infection, stimulated macrophages and epithelial cells secrete IL-6 and IL-1β, which induce Th17 differentiation and promote IL-17A production; IL-17A in turn further stimulates epithelial cells and macrophages to release IL-6 and IL-1β, perpetuating inflammation. Interrupting this cycle through dual targeting of IL-17A and IL-6 could represent an effective therapeutic strategy, though such approaches remain to be experimentally validated.

#### 5.3.3 Dendritic cells as orchestrators of pathogenic T-cell responses

Dendritic cells (DCs) emerge as pivotal orchestrators of mycoplasma VED, linking innate recognition of LAMPs to maladaptive T cell responses. Following *M. pneumoniae* infection, antigen-bearing pulmonary DCs migrate to draining lymph nodes to prime naïve T cells. Crucially, the mode of DC activation determines the subsequent T helper polarization (75). Studies show that *M. pneumoniae*-primed DCs preferentially drive Th2 differentiation characterized by IL-4, IL-5, and IL-13 secretion, promoting eosinophilia, mucus hypersecretion, and airway hyperreactivity—hallmarks of mycoplasma pneumonia immunopathology (76).

While the Th2-promoting capacity of mycoplasma-primed DCs differs from the Th17-dominant response to LAMP vaccination, this apparent dichotomy belies a more complex interaction, as distinct *Mycoplasma pneumoniae* components differentially program DC function. Through TLR2 engagement, LAMPs may drive DCs to secrete high levels of TGF-β and IL-23 while suppressing IL-12, thereby skewing differentiation toward Th17 rather than Th1 lineages (76, 77). Notably, emerging evidence reveals functional crosstalk between Th2 and Th17 pathways: type 2 cytokines can amplify Th17 responses through CD209a-dependent signaling on DCs, whereas IL-17A enhances IL-4 and IL-13 production by innate lymphoid and Th2 cells (78). This reciprocal regulation raises the possibility that DCs mediating mycoplasma VED may coactivate both Th2 and Th17 programs, establishing a complex immunopathological network in which each pathway potentiates the other.

#### 5.3.4 Suppression of protective Th1 responses: lessons from other pathogens

Although Th2 and Th17 responses are key mediators of mycoplasma-driven pathology, Th1 responses characterized by IFN-γ production facilitate bacterial clearance and restrain inflammation (79, 80). The failure to mount robust Th1 immunity in VED thus implicates active suppression mechanisms. Insights from other pathogen models provide useful analogies. In *Mycobacterium tuberculosis* infection, the early secreted antigenic target 6 kDa (ESAT-6) protein programs DCs to suppress IL-12 while promoting IL-23 and IL-1β secretion, favoring Th17 polarization at the expense of protective Th1 responses (77). Likewise, *Neisseria gonorrhoeae* subverts host immunity by selectively suppressing Th1 and Th2 differentiation and enhancing Th17 responses through TGF-β-dependent signaling (81).

These parallels suggest that *M. pneumoniae* LAMPs may function analogously, reprogramming DCs to inhibit IL-12 while augmenting IL-23 production, thereby favoring pathogenic Th17 differentiation over protective Th1 immunity. Experimentally, neutralization of TGF-β during *N. gonorrhoeae* infection redirects immune responses from Th17 toward Th1/Th2, establishing protective memory and accelerating bacterial clearance (81). By extension, mycoplasma vaccine formulations or adjuvants that limit TGF-β signaling or enhance IL-12 production could potentially shift immunity away from pathological Th17 toward protective Th1 responses. However, because Th2 responses themselves contribute to mycoplasma immunopathology, such interventions must be precisely tuned to avoid replacing one detrimental immune bias with another.

#### 5.3.5 An integrated model of VED immunopathology

Collectively, the evidence supports an integrated model in which LAMP-primed DCs serve as central orchestrators of multiple pathogenic pathways. First, TLR2-mediated recognition of LAMPs induces high TGF-β and IL-23 secretion with concurrent IL-12 suppression, promoting Th17 over Th1 differentiation. Second, these same DCs may concurrently favor Th2 polarization through incompletely defined mechanisms, potentially involving CD209a-dependent amplification. Third, Th2 and Th17 effector cells engage in reciprocal cross-regulation: IL-17A enhances type 2 cytokine production, while type 2 cytokines further potentiate Th17 responses. Fourth, both pathways converge on excessive neutrophilic inflammation that, in the absence of effective bacterial clearance, culminates in tissue destruction.

This model generates testable predictions: first, dual inhibition of Th2 and Th17 pathways should provide greater protection than targeting either alone; second, vaccine formulations that promote DC production of IL-12 while limiting TGF-β and IL-23 signaling should favor protective Th1 responses. Importantly, this framework extends beyond IL-17A to encompass the broader cytokine networks that collectively determine whether mycoplasma vaccination confers protection or predisposes to enhanced disease. Future studies must therefore adopt systems-level approaches that interrogate interactions among DCs, T cell subsets, and innate effectors to fully elucidate VED mechanisms and guide rational vaccine design.

### 5.4 The role of B cells and antibodies: protection, regulation, and immune correlates

#### 5.4.1 Antibodies provide partial protection

In contrast to viral pathogens such as dengue, RSV, and measles, where antibody-dependent enhancement (ADE) drives pathology, antibodies against *M. pneumoniae* primarily mediate protection by reducing bacterial burden (40–49). Passive transfer of hyperimmune sera from LAMP-vaccinated mice fails to reproduce VED, excluding antibody-mediated enhancement as a causal mechanism (39). Monoclonal antibodies targeting the P1 adhesin afford partial protection by reducing lung pathology and colonization (39, 83, 84). Nevertheless, humoral immunity alone is insufficient for full protection: heat-denatured dLAMP vaccines, which disrupt conformational epitopes, lose efficacy despite preserved immunogenicity (39), and anti-P1 antibodies diminish but do not eliminate colonization.

#### 5.4.2 Antibodies are necessary but not sufficient: immune correlates remain undefined

Protein-specific IgG antibodies are essential for bacterial clearance, whereas glycolipid-specific antibodies are dispensable (82). B-cell-deficient mice fail to clear infection despite intact cellular immunity, underscoring the necessity of antibody-mediated defense (82). However, individuals harboring pre-existing antibodies from prior infections can still develop pneumonia upon reinfection (17, 62). Notably, schoolchildren with serologic evidence of infection during one epidemic were not protected during the subsequent outbreak (17), and the occurrence of asymptomatic carriers and reinfections demonstrates the limited durability of antibody-based protection (85).

This dissociation between antibody presence and clinical protection indicates that antibody titers alone are inadequate correlates of immunity. Effective protection likely requires: first, adhesin-blocking antibodies with *in vitro* neutralizing activity; second, balanced Th1 rather than pathological Th17/Th2 responses. Validated immune correlates of protection remain undefined, highlighting the need for prospective studies linking pre-challenge immune profiles to clinical outcomes.

#### 5.4.3 B cells as immunoregulators beyond antibody production

B-cell depletion during LAMP vaccination markedly worsened disease upon challenge, producing greater severity and extensive neutrophil infiltration (19). Typical VED lesions feature organized aggregates of CD19^+^ B cells and CD4^+^ T cells; their depletion abolished these structures and replaced them with diffuse neutrophilic consolidation (39). These findings indicate that B cells possess regulatory functions beyond antibody production—most likely through IL-10 secretion that limits excessive Th17 activity. Thus, lipoproteins simultaneously activate and are restrained by B cells, a paradox central to mycoplasma-induced VED.

### 5.5 Implications for vaccine development

A logical question arises if lipoproteins are indeed responsible for VED: why are LAMPs still employed in vaccine research? The resolution lies in the fact that the experimental use of LAMPs serves to dissect VED mechanisms; these are research tools, not proposed clinical candidates (39, 40). Indeed, the critical distinction between lipoproteins and protein antigens as vaccine targets becomes clear when examining their divergent immunological roles. Lipoproteins activate TLR2, which programs dendritic cells to favor pathogenic Th17 over protective Th1 responses and induces IL-17A-driven neutrophilia that mediates VED (40). This mechanism explains why historical vaccines using whole killed bacteria or crude membrane preparations, all of which contained lipoproteins, consistently resulted in disease enhancement rather than protection (29–31, 61). In contrast, antibodies against protein adhesins, particularly P1 and P30, are essential for host defense (82, 84).

The protective potential of protein-based immunity is supported by both functional and experimental evidence. Adhesin-specific antibodies directly block bacterial attachment, the critical first step in pathogenesis, whereas lipoprotein-specific antibodies target membrane components irrelevant to host-pathogen interactions (82). Monoclonal antibodies against P1 inhibit bacterial adhesion and reduce colonization (39, 83), demonstrating that targeting protein epitopes can confer protection. These mechanistic insights indicate that rational vaccine design should utilize recombinant P1 and P30 adhesins maintaining native conformational epitopes, formulated with Th1-promoting adjuvants to avoid the Th17-mediated pathology associated with lipoprotein-containing preparations.

## 6 A rational framework for vaccine design: integrating lessons from immunopathology

The foremost challenge in mycoplasma vaccinology is to dissociate protective immune mechanisms from IL-17A-mediated immunopathology while preserving beneficial B-cell functions. Achieving this balance demands an integrated strategy that goes beyond simply suppressing inflammation and instead actively programs balanced, durable, and protective immunity through coordinated advances in antigen engineering, adjuvant formulation, and delivery platforms.

### 6.1 Engineering the antigen: prioritizing safety and conformational fidelity

Rational antigen redesign offers a direct route to mitigate VED by preserving native structure and immune recognition. Heat-denaturation studies reveal that while linear epitopes may remain immunogenic, disruption of conformational epitopes abolishes protective efficacy (39). These findings highlight that effective immunity against mycoplasma infections relies largely on antibodies recognizing the native three-dimensional structures of key adhesins like P1, P30, and P116, rather than on linear sequences or lipid-associated antibodies (25, 82). Future antigen engineering should therefore focus on stabilizing these conformational epitopes to elicit potent and functionally protective antibody responses.

#### 6.1.1 Structural stabilization through proline substitution and disulfide bond engineering

Building on precedents from viral vaccine design, most notably the prefusion-stabilized SARS-CoV-2 spike protein, similar strategies could benefit mycoplasma antigens (86). For the immunodominant P1 adhesin, computational modeling can identify flexible regions where strategic proline substitutions would rigidify the backbone and lock the molecule into a prefusion-like conformation, preserving neutralizing epitopes. Additionally, engineered disulfide bonds between β-strands in the C-terminal domains of P1 may prevent conformational collapse during expression and immunization, maintaining antigenic fidelity and optimizing immune presentation (87).

#### 6.1.2 Surface charge optimization for targeted immune recognition

Beyond structural stabilization, the electrostatic surface properties of antigens profoundly influence immunogenicity and host interactions. Computational platforms such as Rosetta and PyDock can identify clusters of charged residues on mycoplasma adhesins that facilitate nonspecific interactions with host proteins (88). Targeted substitution of these charged residues with neutral amino acids can be designed to achieve a dual objective: to attenuate undesirable binding to host proteins like complement components or Fc receptors, and to preserve the accessibility of key epitopes for effective immune recognition. Applying this principle to broadly adhesive molecules such as P116 could yield variants that retain effective ciliary adherence yet exhibit reduced erythrocyte agglutination—thereby improving both vaccine specificity and safety (89). Nonetheless, empirical validation is essential to substantiate these computational predictions.

### 6.2 Directing the immune response: harnessing B cell functions while controlling pathogenic T cell responses

Adjuvant choice and delivery platform design play pivotal roles in directing adaptive immune responses. Insights from VED studies in *Mycoplasma* infections reveal that optimal formulations should not merely suppress inflammation but actively promote robust B cell activation and antibody generation, while steering T cell polarization away from pathogenic Th17 responses (39, 83).

#### 6.2.1 Immunomodulatory adjuvants for precision immune regulation

Mechanistic studies underscore that adjuvant selection fundamentally dictates the balance between protective and pathological immunity. Conventional aluminum-based adjuvants favor Th2 and Th17 polarization, which are suboptimal against intracellular or adhesion-dependent pathogens (90, 91). Consequently, a rational transition toward adjuvant systems that preferentially induce Th1 and regulatory responses is warranted.

##### 6.2.1.1 TLR4 agonists

TLR4 agonists can suppress vaccine-enhanced Th2 and Th17 responses while maintaining protective antibody production. Studies with formalin-inactivated RSV vaccines demonstrated that LPS adjuvantation inhibited lung inflammation and promoted opsonophagocytic IgG2a antibodies without compromising viral clearance (92, 93). Monophosphoryl lipid A (MPLA), a detoxified LPS derivative, activates TLR4 to induce strong Th1 responses with minimal inflammatory toxicity. Approved in vaccines such as Cervarix and Shingrix, MPLA enhances both antibody titers and cell-mediated immunity (94). For mycoplasma vaccines, MPLA formulated with adhesins may drive opsonophagocytic antibody responses (IgG2a/IgG2c in mice, IgG1 in humans). It is plausible that TLR4 activation promotes regulatory B cell differentiation via IL-10 production, thereby potentially counteracting Th17-mediated immunopathology.

##### 6.2.1.2 CpG oligodeoxynucleotides as an adjuvant

One notable advantage of CpG-ODN is its demonstrated capacity to prevent VED in preclinical models (95). In RSV vaccine studies, CpG supplementation effectively redirected detrimental Th2-biased responses toward protective Th1 immunity, thereby reducing lung inflammation (96). CpG-ODNs activate B cells and plasmacytoid dendritic cells, inducing vigorous Th1-type responses—an effect exemplified by the licensed Heplisav-B vaccine (97, 98). In the context of mycoplasma vaccines, intranasal CpG-ODN delivery could stimulate mucosal immunity and foster the development of tissue-resident memory cells at colonization sites, while enhancing antibody affinity maturation through prolonged germinal center activity. Furthermore, synergistic formulations combining CpG-ODN with MPLA in nanoemulsion platforms hold promise for maximizing Th1 polarization while minimizing reactogenicity.

##### 6.2.1.3 IL-10 as an adjuvant: a double-edged sword in balancing inflammatory responses

IL-10, a pivotal immunoregulatory cytokine, exerts a dual and context-dependent influence on vaccine-induced immune responses (99). It functions to suppress excessive inflammation and mitigate immunopathological damage. In a Leishmania major infection model, Gonzalez-Lombana et al. demonstrated that IL-10 deficiency led to markedly elevated IL-17 production and intense neutrophil infiltration, culminating in severe tissue injury (100). These findings suggest that controlled incorporation of IL-10 into mycoplasma vaccine formulations could temper hyperactive Th17 responses and reduce vaccine-associated inflammatory sequelae. However, the timing and dosage of IL-10 administration are critical determinants of its immunological outcome. Overexpression or excessive supplementation may dampen protective immunity, thereby diminishing vaccine efficacy. Striking an optimal balance between enhancing protection and minimizing immunopathology thus remains a central challenge for leveraging IL-10 as an adjuvant in mycoplasma vaccines, necessitating further mechanistic and translational investigation.

##### 6.2.1.4 Vitamin D3 as adjuvant: a novel strategy for modulating Th17/Treg/Breg balance

Vitamin D3 has gained attention as a promising immunomodulatory adjuvant capable of fine-tuning adaptive immune responses. Emerging evidence indicates that vitamin D3 priming reprograms dendritic cells to suppress Th17 differentiation while promoting regulatory T cell (Treg) and regulatory B cell (Breg) cell induction, even within pro-inflammatory, neutrophil-rich environments. This reprogramming is mediated through downregulation of Th17-polarizing cytokines such as IL-23, coupled with increased generation of IL-10–secreting Tregs and Bregs (101, 102). In the context of mycoplasma vaccination, such modulation offers a double-edged potential: moderate vitamin D3 supplementation may mitigate excessive vaccine-induced inflammation, yet its inhibitory effect on Th17 polarization could attenuate protective immunity dependent on Th17-mediated defense mechanisms. The synergistic or antagonistic interactions between vitamin D3 and conventional adjuvants remain poorly characterized. Determining its optimal dosing, formulation compatibility, and mechanistic interplay with other immunostimulants warrants systematic experimental validation. It should also be noted that most current data originate from autoimmune and fungal infection models, and thus, vitamin D3’s efficacy in modulating protective responses against mycoplasma infection remains to be elucidated.

#### 6.2.2 Particulate delivery systems for biomimetic immune activation

Particulate delivery systems that emulate pathogen size and architecture have proven effective in amplifying immunogenicity by enhancing antigen presentation and innate immune activation. Key biophysical parameters, including particle size, geometry, surface charge, and antigen density, can be engineered to optimize B-cell activation while minimizing undesirable inflammatory responses.

##### 6.2.2.1 Self-assembling protein nanoparticles

Nanoplatforms such as ferritin and I3–01 nanoparticles present mycoplasma adhesins in highly ordered, multivalent arrays, significantly augmenting B-cell receptor cross-linking (103, 104). This strategy, which has shown marked success in influenza and HIV vaccines (104), could be applied to the mycoplasma P1 adhesin. Fusion of P1 to ferritin may yield a 24-subunit nanoparticle capable of displaying P1 in its native trimeric conformation, potentially amplifying immunogenicity by 10- to 100-fold. Such particulate vaccines typically elicit balanced Th1/Th2 responses with minimal Th17 bias, thereby reducing the risk of VED. The I3–01 platform, featuring a 60-subunit icosahedral scaffold, offers even higher antigen valency and has demonstrated superior immunogenicity with the RSV F protein, highlighting its potential as a versatile platform for multivalent *Mycoplasma* vaccines.

##### 6.2.2.2 Liposomes and lipid nanoparticles

Building on advances in LNP technology accelerated by mRNA vaccines (105), novel delivery strategies for mycoplasma antigens are now emerging. A promising approach involves the use of cationic liposomes to encapsulate protein antigens, enabling their direct delivery to antigen-presenting cells while also promoting B cell receptor cross-linking through surface-displayed antigens, all while potentially avoiding excessive inflammation (106). Beyond proteins, ionizable LNPs offer a pathway for delivering mRNA encoding key adhesins like P1 or P116. This approach ensures the endogenous expression of antigens in their native conformation, which is critical for eliciting protective antibodies. Furthermore, the versatility of LNPs allows for the co-encapsulation of immunomodulators, such as IL-10 or vitamin D3 analogs, to potentially foster a pro-regulatory microenvironment at the injection site and mitigate adverse inflammation.

##### 6.2.2.3 Polymeric microparticles

PLGA microparticles enable controlled, sequential release of antigens and immunomodulators. By engineering particles with distinct degradation kinetics, a single injection could elicit both priming and boosting immune responses (107). In the context of mycoplasma vaccines, this approach could temporally separate early antigen release, driving initial antibody induction, from delayed release of immunoregulatory agents such as IL-10 or vitamin D_3_, ensuring that protective immunity is established before regulatory mechanisms become dominant. Surface modification with targeting ligands (e.g., anti-DEC205) could further direct antigen uptake to specific dendritic cell subsets while avoiding those that promote Th17 polarization.

The rational design of diverse particulate systems, including protein nanoparticles, lipid-based carriers, and polymeric microparticles, enables precise control over antigen presentation and immune modulation. By leveraging biomimetic properties and tunable release kinetics, such platforms offer promising avenues to enhance vaccine efficacy against mycoplasma infections while minimizing the risk of vaccine-enhanced disease.

## 7 Conclusion and future perspectives

Developing safe and effective human mycoplasma vaccines requires navigating the IL-17A paradox. Central insights, encompassing the pathogenic contribution of bacterial lipoproteins, the IL-17A–neutrophil axis, and the regulatory functions of B cells, form a mechanistic foundation for rational vaccine design. Achieving the appropriate balance is critical: eliciting robust conformation-specific antibodies and sustaining Breg activity while preventing Th2/Th17 skewing that compromises protection. Understanding this dual nature of immune responses in mycoplasma-associated VED not only addresses a longstanding barrier but also informs vaccine development for other mucosal pathogens, where immunity must delicately balance defense and tolerance.
